# Artificial intelligence–based tools to address current gaps in transition to adult care for youth with chronic surgical conditions: a perspective piece

**DOI:** 10.1136/wjps-2025-001099

**Published:** 2026-07-07

**Authors:** Siena O’Neill, Elena Guadagno, Esli Osmanlliu

**Affiliations:** 1 Department of Anatomy and Cell Biology, McGill University Faculty of Science, Montreal, Québec, Canada; 2 Department of Pediatric Surgery, Harvey E. Beardmore Division of Pediatric Surgery, Montreal Children’s Hospital, Montreal, Québec, Canada; 3 Department of Pediatrics, Division of Emergency Medicine, Montreal Children’s Hospital, McGill University Faculty of Medicine and Health Sciences, Montreal, Québec, Canada; 4 Centre for Outcomes Research and Evaluation, RI-MUHC, Montreal, Québec, Canada

**Keywords:** Adolescent Health, Education, Medical, Pediatrics, Technology

## Fictional clinical vignette

Jane is a 17-year-old patient with biliary atresia (BA). At her last appointment, her doctor began explaining what will happen when she transfers to adult care this coming fall—Jane knew this would happen eventually, but she did not realize it would happen so soon. Her doctor has always told her about a time when everything would be taken care of at the adult hospital, but the conversation always ended there. Now, Jane feels like she has somehow fallen behind; what if there were more she could have done to prepare? She does not know how to renew a prescription or schedule an appointment. She feels like she barely understands her own condition—she knows about her cirrhosis and low platelets, but not about the meaning behind it all, not really. How will she be able to manage all of this on her own?

## Transition to adult care: a critical step in promoting the health of youth with chronic surgical conditions

Adolescence is a period filled with social, emotional, and physical change, calling for increased autonomy and self-reliance in preparation for adulthood. For youth living with a chronic surgical condition, adolescence becomes further complicated; the sudden need to navigate a new healthcare system on their own makes the proper planning and execution of the transition to adult care essential.^1^

Healthcare transition is defined as the process of preparing a patient for the shift to adult care, while transfer refers to the official switch from pediatric to adult healthcare teams within the transition process.^2^ Starting this process early, and ensuring adequate communication, support, and education throughout is key to maintaining continuity of care, preventing patient disengagement, and mitigating negative health outcomes.^1–4^ Currently, many areas of transition care are lacking, and patients are often left navigating the process on their own, in part due to constrained capacity among healthcare teams.^2–4^ An array of artificial intelligence (AI) tools can help bridge these gaps, facilitating the transition process while re-humanizing patient-provider interactions.

AI models can employ machine learning (ML), deep learning (DL), and/or natural language processing (NLP) to perform conversational (analyzing and replicating human language), predictive (classifying images and predicting outcomes), and generative tasks (producing new text and images).^5 6^ In healthcare, conversational AI can be used to facilitate healthcare interactions, predictive AI to support diagnostic and prognostic tasks, such as anticipating the need for surgery, and generative AI to create disease-specific educational material for patients.

Such applications of AI can improve partnerships between patients and their healthcare teams, amplify patient voices, promote engagement, and strengthen relationships with providers.

This perspective is informed by lived experience navigating chronic surgical care as a patient and parent (authors SO and EG, respectively), a review of adolescent healthcare transition literature, and an environmental scan of apps and online tools supporting this process. Drawing on the authors’ personal experiences, our paper lends itself to discussing BA care as an illustrative example of the healthcare transition process.

We aim to identify common barriers to successful healthcare transitions for chronic surgical conditions and propose a conceptual framework for a series of corresponding AI-based solutions.

## Current barriers in adult care transitions

We reviewed medical literature in Medline (Ovid) addressing the transition of adolescent surgical patients to adult healthcare from a non-clinical perspective. Included studies discussed current practices or offered recommendations for future transition care in the context of chronic illness, surgical conditions, or BA specifically (See online supplemental appendix A for search strategy).

10.1136/wjps-2025-001099.supp1 Supplementary data

Additionally, given the increasing use of digital health technologies by patients, an informal search was undertaken using the Google Web search engine and the App Store (Canada) to identify healthcare transition resources that are readily available to patients.^7^

As a result of the focused literature review, we selected seven articles examining care transitions among patients with chronic conditions, based on relevance and publication between 2014 and 2025, that discussed either the status of transition to adult care or suggestions for future practices. Of these, six focused on surgical conditions, including one article that specifically addressed healthcare transition in patients with BA. Several overlapping topics were identified across these seven papers, either framed as recommendations for improving care (*i.e.*, transition planning *should* begin early) or deficits in current standards of care (*i.e.*, not enough pre-transition discussions *are* taking place).^2 3^ These topics fell under the broader categories of communication, psychosocial support, patient and provider education, and institutional policies. We extracted topic mentions from each article and tallied how many times each topic appeared, whether referenced generally or definitively identified as a gap in care. A total of 16 healthcare transition topics were identified across all seven articles. Topics relating to hospital policies and institutional aspects surrounding transition care (*i.e.*, questionnaire implementation, age of transition, *etc.*) were the most frequently discussed in the selected papers. However, we focused on less frequently reported gaps that are highly relevant to the care experience of patients and caregivers: communication, support, and education.

Following the online search for patient resources, we selected 11 results, including websites, apps, and one video. Most of these resources were not disease-specific or particular to pediatric surgery, and their content ranged from resource links on hospital websites to transition readiness quizzes in interactive apps. The video was the only resource specific to BA. Most of the features included in these sources failed to adequately address the gaps that were identified in this brief literature review, as well as in the author’s experience as a patient with BA. Notably, none of these resources used AI technologies to enhance any of their features. This becomes an area where conversational, predictive, and generative AI can potentially assist in creating a more personalized and humanized transition experience, emphasizing patient-provider communication, psychosocial support, and disease education.

### Late initiation of the transition process

In the realm of *communication*, the gaps identified in our analysis of the selected articles concerned failure to initiate the transition process at a young enough age, and lack of preparatory discussions being held between the patient, family, and provider prior to the transfer of care.^1–3^ It is recommended, for example, in BA care, that a transition program be initiated at the age of 12 to allow the patients to gradually take on new responsibilities in managing their care; however, this often is not the case.^1–4^ This goes hand in hand with the frequently absent or inadequate patient-provider discussions that occur in the years leading up to transfer, with the proposed goal of informing the patient and family what is expected of them throughout the transition process.^2 3^

### Lacking psychosocial monitoring and support

Patients with chronic surgical conditions are more likely to experience poor mental health and a reduced quality of life than their healthy peers, which becomes particularly pronounced during adolescence, where healthcare transition occurs in tandem with many other developmental and social changes in the patient’s life.^1 2 4 8^ *Psychosocial support*, including mental health resources, peer support groups, and increased guidance and involvement from the healthcare team, is crucial for these young adults. However, such support frequently lacks in current models of transitional care.^2 4 8^

### Insufficient patient and provider education

Patient *education*, while not discussed explicitly as a deficit in care, involves providers ensuring that patients know enough about their own diagnosis and medical history as they become more independent, since many major events in their care likely occurred before they were old enough to understand.^1 4^ Adult providers could also benefit from improved transition education—an aspect of care often overlooked—particularly with respect to the intricacies of their patient’s diagnosis and surgical history, as they often lack experience treating complex pediatric surgical patients and can be ill-equipped to care for them.^2 3^

## Opportunities afforded by health AI technologies

**Figure 1:**
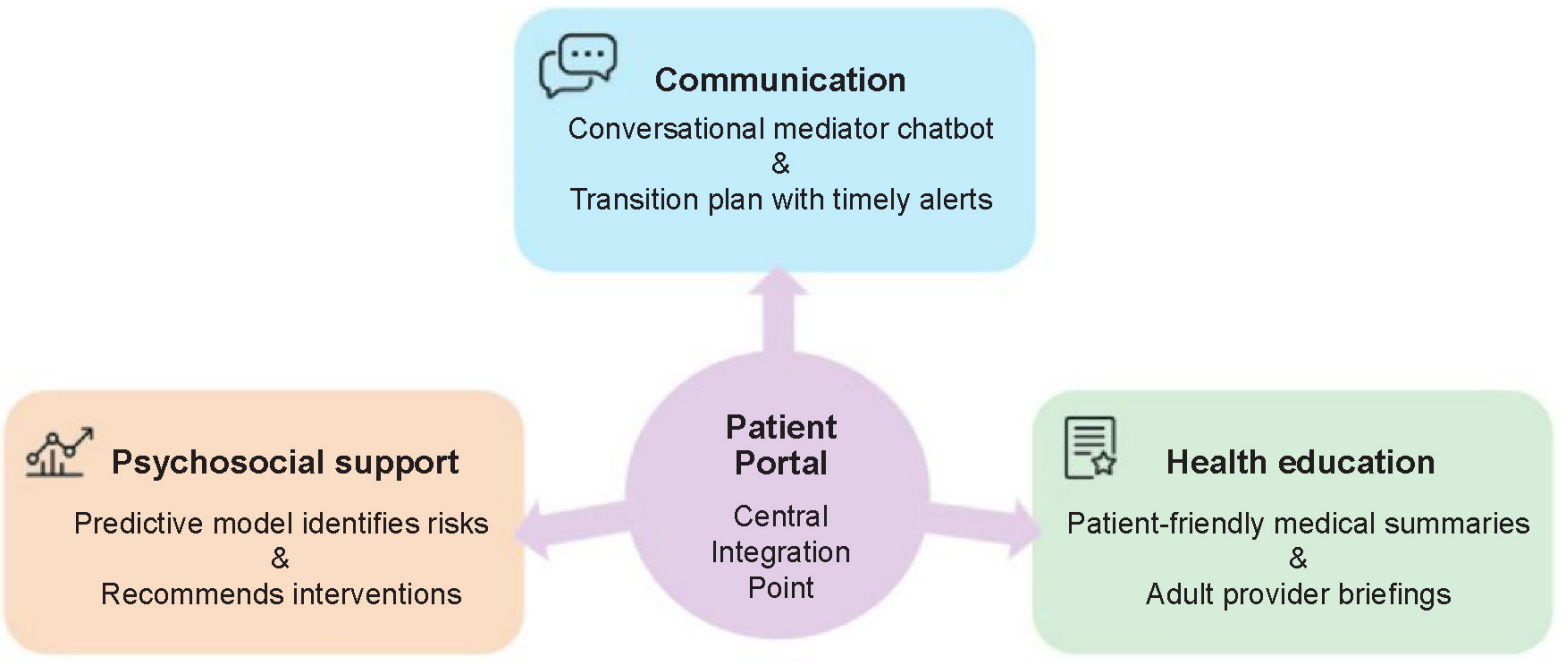
Artificial intelligence–based tools supporting transition to adult care for youth with chronic surgical conditions.

Given the lack of AI-based tools available for the transition to adult care, we propose the following concepts for AI-driven features as components of a comprehensive patient portal to aid in transition communication, support, and education (figure 1).

### Communication

To address communication gaps between patients and providers, an AI chatbot can act as a conversational mediator, bridging language barriers and power dynamics to facilitate preparatory discussions regarding transition planning.^9^ This could begin with conversation topics suggested by the chatbot based on the patient’s circumstances, age, and capabilities, and extend to using this NLP-driven conversational AI model to interpret the words of the patient and provider in a way that both parties will understand, so as to re-humanize healthcare interactions and strengthen communication.^5 9^ Additionally, to encourage providers to begin transition planning early, a large language model (LLM), which incorporates both generative and language processing abilities, can be used to assess the patient’s readiness to commence the transition process by administering and analyzing the results of a standardized questionnaire. When the patient is deemed ready, the LLM can generate the steps of an individualized transition timeline, notifying both the patient and provider each time a new age-appropriate preparatory discussion should be held.

### Psychosocial support

Predictive AI models can be used to assess and predict the state of a patient’s psychosocial health through the use of patient-reported outcomes (PROs) as both input and output.^9^ Using PRO data relating to a patient’s perceived quality of life, transition readiness, and mental well-being, in conjunction with clinical history and patient characteristics, this model would use ML to recognize patterns and identify the underlying cause of a patient’s recent emotional state or health-related behaviors, such as anxiety or non-adherence resulting from an inadequate support system.^5^ Knowing this, healthcare providers can increase the frequency of visits, prioritize mental health resources, and adapt care plans to properly meet their patients’ needs. The results could also be fed into a generative AI model to provide tailored resources and help design a personalized intervention plan. Conversely, PROs and clinical data can be entered into the same predictive model in order to anticipate psychosocial PROs following a health intervention or new step in the transition plan, allowing clinicians to be aware of the impact a given intervention will have on a patient and preventing negative psychosocial health outcomes from going undetected.^10^

### Education

LLMs embedded into electronic health records can create simplified summaries of complex medical records and appointment notes, rendering their content searchable for both patients and future adult providers. This could improve health literacy and allow patients to have easier access to their medical history and the details of their condition, all of which may previously have been directed toward their caregivers. Access to a personal medical history search tool, or to a plain-language explanation of their diagnosis, can empower patients and allow them to feel more confident in managing their own care. Before a patient officially transfers care, this summary tool can highlight key events in their history and important notes from pediatric providers to give the adult practitioner a better picture of the patient’s condition. This is especially useful for educating providers who have limited experience in treating other patients with a similar condition. Selected personal information from the patient’s profile can be incorporated to provide the practitioner with a whole-person view of the patient prior to their first encounter. Adult providers can also use these features alongside the corresponding original health records to review a patient’s history with them during an appointment.

To ensure that these AI-based interventions are engaging, yield positive results, and offer the most benefit to the adolescent patient populations that they are serving, these tools must be co-designed with youth and trained on inclusive and diverse data.^10 11^ Guidelines such as Pediatrics EthicAl Recommendations List for AI (PEARL-AI) and ACCEPT-AI provide guidance on key principles for developing successful child-centric and youth-centric health AI tools.^12 13^

### Limitations of AI

We acknowledge that the above-mentioned AI solutions may not be widely applicable and, in practice, not as beneficial as anticipated. With the use of any AI model, algorithmic bias can arise from improper data generalizations and a lack of inclusion of the representative populations in both the design and training processes.^12^ Failing to train the model on diverse datasets can result in the amplification of societal biases, making the AI-generated content impertinent or even harmful in certain cases. Concerns of confidentiality and consent or assent would also arise when dealing with pediatric data, justifying measures that ensure adequate privacy and data protection.^12^ In addition, for the proper application of the AI summary tools, all patient files would have to be digitized to mitigate the risk of excluding pertinent medical information. The capacities of generative AI are limited, and the accuracy and nuance that would be sought after in the summaries of medical records and appointment notes may not, in reality, be achieved, and a whole-person perspective may not come through. Such platforms should also acknowledge and support the patients’ right to digital sobriety by promoting the use of AI-based tools when most pertinent and where evidence indicates clinical benefits.^14^

## Conclusion

Adolescents living with chronic surgical conditions are often not receiving the care and support that they require during their transition to adult healthcare. Our analysis of the included articles highlights the deficits that are still present in transition care, including a lack of patient-provider communication, insufficient or absent psychosocial support, and the need for proper health education for both patients and providers.^2 4^ Additionally, among the current transition resources that were identified, none employed health AI technologies, and most could benefit from an improvement in disease specificity and patient engagement. In order to supplement these gaps in care and promote patient involvement, the AI-driven tools suggested by the authors can be used to perform conversational, predictive, and generative tasks. To improve communication and ensure transition planning begins early, an AI chatbot can facilitate healthcare discussions, while interactions with an LLM can be used to evaluate a patient’s readiness and generate an individualized transition plan. To address the patient’s psychosocial health, a predictive AI model can ‘diagnose’ the source of current challenges and predict future PROs, suggesting appropriate resources and providing a tailored intervention plan. In terms of education, a generative AI summary tool can improve health literacy by making complex medical records more accessible to patients and educating adult providers about lesser-known pediatric surgical conditions. Ultimately, AI can be employed in healthcare settings to establish meaningful patient partnerships, allowing adolescent patients to feel more confident, involved, and better supported in their care.
