# Supplementary material for: Artificial intelligence–based tools to address current gaps in transition to adult care for youth with chronic surgical conditions: a perspective piece
Source: World J Pediatr Surg. 2026 Jul 7;9(4):e001099. doi: 10.1136/wjps-2025-001099 (PMC13358331; doi:10.1136/wjps-2025-001099)
Supplement: Supplementary data [file wjps-9-4-s001.pdf]

Appendix A: Search Strategy
Medline [Ovid] (August 17, 2025)
Ovid MEDLINE(R) and Epub Ahead of Print, In-Process & Other Non-Indexed Citations and Daily <1946 to August 15, 2025>

1	*Transition to Adult Care/	2239
2	((transition* adj2 adult*) or (transition* adj care*)).ti,kf.	3780
3	((transition* adj2 adult*) or (transition* adj care*)).ab. /freq=2	1916
4	or/1-3	5991
5	Biliary Atresia/	3925
6	exp Bile Ducts/	52786
7	limit 6 to yr="1966 - 1986"	12162
8	(biliar* adj3 (atres* or atroph*)).tw,kf.	5950
9	Portoenterostomy, Hepatic/	990
10	(kasai or portoenterostom*).tw,kf.	1924
11	5 or (or/7-10)	18558
12	exp *Specialties, Surgical/	182226
13	exp *Surgical Procedures, Operative/	2421475
14	su.fs.	2405709
15	(surger* or surgical* or operati* or transplant*).ti,kf. or (surger* or surgical* or operati* or reoperat* or transplant* or laparoscop* or laparotom*).ab. /freq=3	1974409
16	or/12-15	4381685
17	11 or 16	4390194
18	4 and 17	396
19	adolescent/ or Adolescent Medicine/ or young adult/	2884895
20	(adolesc* or juvenile* or teen* or youth* or pubescen* or preadolesc* or prepubesc* or preteen* or (young adj1 adult*)).tw,kf.	721799
21	or/19-20	3159189
22	18 and 21	237
23	(transition* adj2 (care or healthcare* or surg*) adj2 (adolesc* or paediatr* or pediatr* or teen* or youth* or adul*)).ti.	331
24	(transition* adj1 (care or healthcare*) adj2 (adolesc* or paediatr* or pediatr* or teen* or youth* or adul*)).ab. /freq=2	44
25	(transition* and adolesc* and adul* and youth*).kf.	21
26	or/22-25	591
27	remove duplicates from 26	591
28	limit 27 to yr="2014 -Current"	488
